# p130Cas scaffold protein regulates ErbB2 stability by altering breast cancer cell sensitivity to autophagy

**DOI:** 10.18632/oncotarget.6710

**Published:** 2015-12-21

**Authors:** Brigitte Bisaro, Marianna Sciortino, Shana Colombo, Maria Pilar Camacho Leal, Andrea Costamagna, Isabella Castellano, Filippo Montemurro, Valentina Rossi, Giorgio Valabrega, Emilia Turco, Paola Defilippi, Sara Cabodi

**Affiliations:** ^1^ Department of Biotechnology and Health Sciences, University of Torino, Torino, Italy; ^2^ Department of Medical Sciences, University of Torino, Torino, Italy; ^3^ Investigative Clinical Oncology (INCO), Fondazione del Piemonte per l'Oncologia (FPO)-Candiolo Cancer Center (IRCCs), Torino, Italy; ^4^ Department of Oncology, University of Torino, Torino, Italy

**Keywords:** p130Cas, ErbB2, autophagy, breast cancer resistance

## Abstract

Overexpression of the ErbB2/HER2 receptor tyrosine kinase occurs in up to 20% of human breast cancers and correlates with aggressive disease. Several efficacious targeted therapies, including antibodies and kinase inhibitors, have been developed but the occurring of resistance to these agents is often observed. New therapeutic agents targeting the endocytic recycling and intracellular trafficking of membrane in tumor cells overexpressing ErbB2 are actually in clinical development. Nevertheless the mechanisms underlying ErbB2 downregulation are still obscure. We have previously demonstrated that the overexpression of the p130Cas adaptor protein in ErbB2 positive breast cancer, promotes tumor aggressiveness and progression. Here we demonstrate that lowering p130Cas expression in breast cancer cells is sufficient to induce ErbB2 degradation by autophagy. Conversely, p130Cas overexpression protects ErbB2 from degradation by autophagy. Furthermore, this autophagy-dependent preferential degradation of ErbB2 in absence of p130Cas is due to an increased ErbB2 ubiquitination. Indeed, the overexpression of p130Cas impairs ErbB2 ubiquitination by inhibiting the binding of Cbl and CHIP E3 ligases to ErbB2. Finally, our results indicate that p130Cas-dependent ErbB2 protection from degradation by autophagy may alter the sensitivity to the humanized monoclonal antibody trastuzumab. Consistently, in human ErbB2 positive breast cancers that develop resistance to trastuzumab, p130Cas expression is significantly increased suggesting that elevated levels of p130Cas can be involved in trastuzumab resistance.

## INTRODUCTION

Molecular and clinical studies indicate that ErbB2 has important implications in tumor etiology and progression. Overexpression of ErbB2 (Her2/Neu), is involved in the pathogenesis of nearly 20–30% of invasive breast cancers and is associated with an aggressive phenotype. Although ErbB2 overexpression identifies patients who are likely to respond to therapy with trastuzumab, not all patients benefit from treatment. Approximately 15% of patients relapse after therapy due to de novo or acquired resistance [1–3]. Therefore, intense investigations are necessary to understand the factors that contribute to the resistance and to identify therapeutic strategies to overcome the resistance. Several mechanisms have been proposed for the acquirement of resistance including the poor internalization of ErbB2 resulting in a long half-life at the plasma membrane [4–7]. Although it has been shown that Hsp90 inhibition can induce ErbB2 ubiquitination followed by its downregulation [8, 9], the mechanisms underlying ErbB2 downregulation are still obscure.

p130Cas is a signaling molecule involved in the linkage of actin cytoskeleton to the extracellular matrix during cell migration, cell invasion and cell transformation. p130Cas protein has been described as a major player in the cross-talk between EGF Receptor and integrins [10]. Due to its modular structure, p130Cas has been shown to play a crucial role in signaling originating from many amplified or mutated oncogenes, by undergoing hyperphosphorylation and association with multiple signaling partners required for transformation [11–13].

The overexpression of p130Cas in the mammary gland leads to hyperplasia and delayed involution but does not promote tumorigenesis [14]. Double transgenic mice originated by crossing MMTV-p130Cas and MMTV-NeuT mice, which express the oncogenic form of the rat *neu* gene, homologous to human ErbB2, showed an accelerated onset of mammary tumor formation. Moreover, the analysis of human breast samples revealed that tumors overexpressing both p130Cas and ErbB2 are characterized by an elevated proliferation index [14]. Our previous data demonstrated that p130Cas is an essential transducer element in ErbB2 transformation and progression showing that p130Cas is necessary for ErbB2-dependent foci formation, anchorage-independent growth, *in vivo* tumor growth and metastatization [15]. Moreover, we have reported that p130Cas over-expression promotes ErbB2-dependent invasion in three-dimensional (3D) cultures of human mammary epithelial cells and we have identified the gene expression changes underlying this invasive behavior [16, 17].

Moreover, p130Cas has been proposed as a crucial modulator of both anti-estrogen and adriamycin resistance [18, 19].

Here we demonstrate that in breast cancer cells overexpressing ErbB2, p130Cas protects ErbB2 from autophagy-mediated degradation by interfering with its ubiquitination. Moreover, changes on the receptor ubiquitination caused by modulation of p130Cas expression leads to expression of different types of autophagic markers, suggesting a link between ErbB2 degradation and autophagy in a p130Cas-dependent manner. Here we show for the first time that high levels of p130Cas expression might be crucial to promote resistance to trastuzumab treatment by protecting ErbB2 from degradation.

## RESULTS

### Modulation of p130Cas expression interferes with ErbB2 protein stability

**Figure 1:**
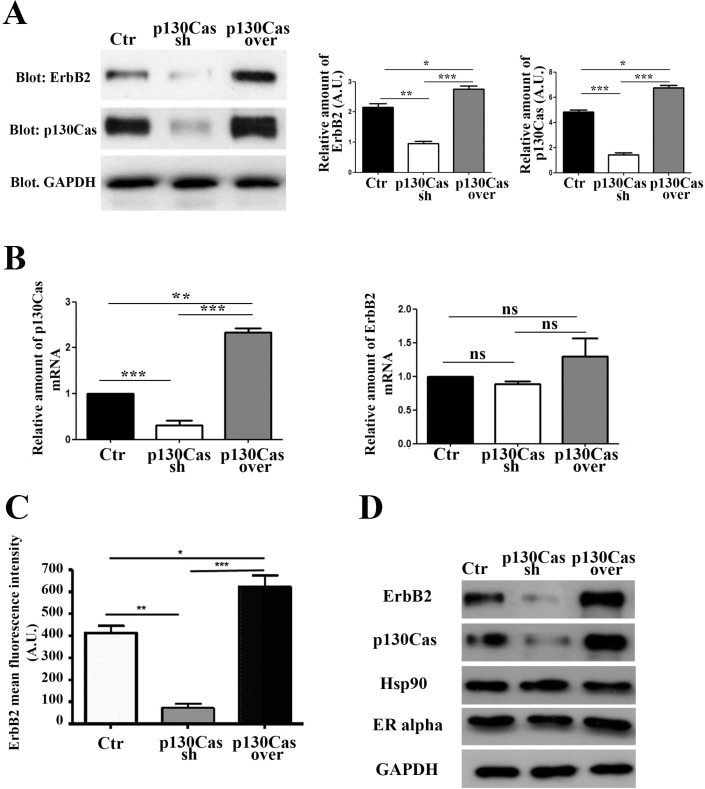
Modulation of p130Cas expression specifically affects ErbB2 expression (**A**) Left panel: Total cell lysates of BT474 cells infected with lentiviral vectors to silence (Cas sh) or overexpress p130Cas (Cas over) were blotted with p130Cas and ErbB2 antibodies. GAPDH was used as loading control. Right panel: Histograms show ErbB2 and p130Cas levels, normalized to GAPDH. Bars represent the means ± SEM of three independent experiments (*p < 0.05; **p < 0.01; ***p < 0.001). (**B**) qRT-PCR analysis of p130Cas mRNA (left panel) and ErbB2 mRNAs (right panel) expression in from cells as in (A). Quantification of results from three independent experiments is shown (ns: not significant; **p < 0.01: ***p < 0.001). (**C**) Histograms show the expression of cell membrane ErbB2 evaluated by FACS analysis on control, p130Cas silenced and p130Cas overexpressing BT474 cells. Bars represent the means ± SEM of three independent experiments (*p < 0.05; **p < 0.01; ***p < 0.001). (**D**) Total cell lysates obtained from cells as in (A) were probed with antibodies to ErbB2, p130Cas, Hsp90 and ER alpha and normalized with GAPDH.

To investigate the relevance of the modulation of p130Cas expression in the control of ErbB2 stability we used, as an experimental model, ErbB2 positive BT474 breast cancer cells. We infected cells with lentiviruses expressing either p130Cas shRNAs or scramble control shRNA sequences, and lentiviruses overexpressing p130Cas with related control vectors. Within 48 hours, p130Cas expression was effectively silenced by about 80% compared to cells infected with scramble sequences, while p130Cas overexpression resulted in about 30–40% increase of protein expression compared to control infected cells (Figure 1A). Interestingly, when we evaluated ErbB2 expression in these cell lysates, we found that p130Cas expression modulation results in changes of ErbB2 expression levels. Indeed, lowering p130Cas expression in BT474 cells (Figure 1A) is sufficient to cause ErbB2 downregulation. The same results were obtained by performing experiments in ErbB2 positive breast cancer cell line SKBR3, further supporting the expression correlation between ErbB2 and p130Cas (Supplementary Figure 1A). To exclude that the ErbB2 downregulation is an off-target effect of sh-p130Cas sequence, we tested four different sequences and we confirmed that lowering p130Cas expression results in ErbB2 downregulation (Supplementary Figure 1B). Consistently, overexpression of p130Cas leads to an increase of ErbB2 expression (Figure 1A). These changes in ErbB2 expression upon modulation of p130Cas expression, were not dependent on alterations of HER2 gene transcription as shown in Figure 1B, (right panel) but rather to its availability on the cell membrane as demonstrated by FACS analysis (Figure 1C). In addition, the alterations of ErbB2 expression upon modulation of p130Cas expression were highly specific, since no expression changes were observed for Hsp90 and ER alpha (Figure 1D).

Therefore, these data indicate that modulation of p130Cas expression in breast cancer cells is sufficient to strongly affect ErbB2 expression.

### p130Cas silencing drives proteasome independent-ErbB2 degradation

Little attention has been paid to the role of ErbB2 degradation in cancers, although when compromised, it may lead to increased ErbB2 levels and activity. Several studies have shown that endocytic downregulation of ErbB2 is impaired in cancer cells although there is poor understanding of how this is achieved [4, 20]. It was recently demonstrated that treatment of ErbB2 positive SKBR3 and BT474 breast cancer cell lines with proteasome inhibitor causes a 50% downregulation of ErbB2 protein expression ([21] and Supplementary Figure 2), indicating that ErbB2 degradation is proteasome independent. In addition, p130Cas has been recently described to regulate cell sensitivity to proteasome inhibition [22].

**Figure 2:**
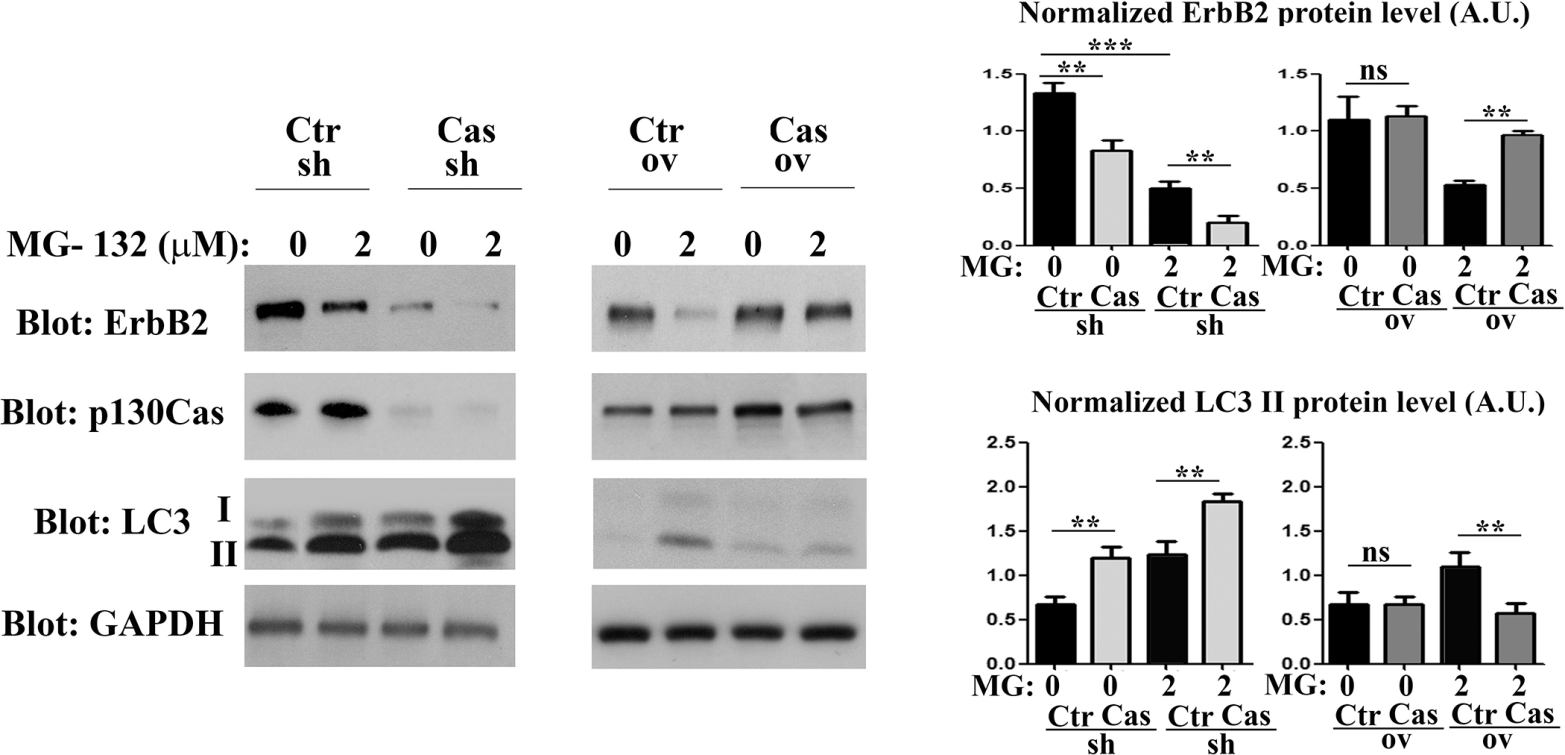
p130Cas expression prevents proteosome-independent ErbB2 degradation Left panel: BT474 silenced for or overexpressing p130Cas and relative controls were treated for 16 hours with MG132 (2 μM). Protein extracts were then blotted with antibodies against ErbB2, p130Cas and LC3. GAPDH was used as loading control. Right panel: Histograms show ErbB2 and LC3-II levels, normalized to GAPDH. Bars represent the means ± SEM of five independent experiments (ns: not significant; **p < 0.01; ***p < 0.001).

To understand whether ErbB2 altered protein levels, as a consequence of modulation of p130Cas expression, implicate proteasome activity, we treated p130Cas silenced, p130Cas overexpressing and relative BT474 control cells for 16 hours with the proteasome inhibitor MG132. As shown in Figure 2, proteasome inhibition induces ErbB2 degradation in control cells, as previously demonstrated. However, we observed a significant increased degradation of ErbB2 in p130Cas silenced cells compared to control cells following treatment with 2 μM of MG132 for 16 hours, while overexpression of p130Cas minimizes the degradation of ErbB2 upon proteasome inhibition. Interestingly, these data indicate that lowering p130Cas expression can lead to further degradation of ErbB2, while the overexpression of p130Cas protects ErbB2 from degradation. Moreover, treatment of p130Cas silenced and relative BT474 control cells with cycloheximide with or without MG132, indicates that ErbB2 degradation is prompted in p130Cas-silenced conditions (Supplementary Figure 3).

It was previously demonstrated that ErbB2 degradation upon proteasome inhibition leads to the formation of cytosolic aggregates directed to lysosomal degradation [23]. Consistently, the treatment of control (Ctr) cells with MG132 induced a reduction of ErbB2 level and an increased amount of the autophagic markers LC3-II (Figure 2 left and right panels), indicating that the induction of autophagy correlates with decreased ErbB2 protein levels. Noteworthily, the downregulation of p130Cas is sufficient to lower ErbB2 expression and to enhance the conversion of LC3-I to its lipidated form LC3-II respect to the control and to the p130Cas overexpressing cells, suggesting that p130Cas-dependent ErbB2 degradation is due to an increased activation of the autophagic process. These experiments were also performed in SKBR3 cell lines showing the same results (Supplementary Figure 4A and 4B).

Most interestingly, high levels of p130Cas expression that protect ErbB2 from degradation correlate with low expression of the autophagy marker LC3-II independently of proteasome inhibition. These data indicate that in ErbB2 positive cells silenced for p130Cas and treated with MG132, p130Cas inhibition leads to a stronger activation of the autophagic flux leading to increased ErbB2 degradation whereas p130Cas overexpression is sufficient to impair autophagy thereby preventing ErbB2 degradation.

### p130Cas expression level alters sensitivity of breast cancer cells to autophagy

**Figure 3:**
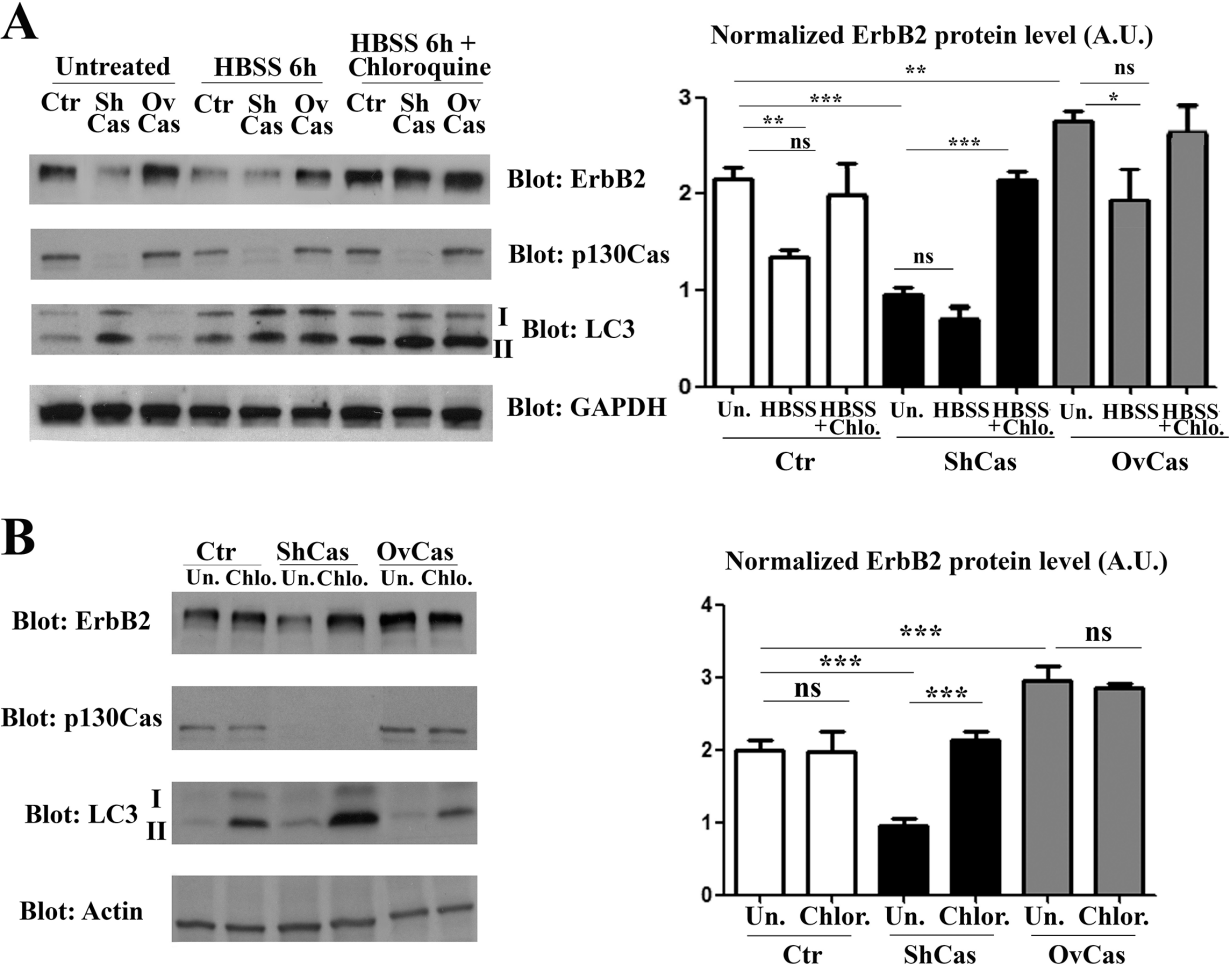
p130Cas protects ErbB2 from autophagy-dependent degradation (**A**) Left panel: Extracts from p130Cas silenced, overexpressing and control (Ctr) BT474 cells cultured for 6 hours in HBSS in absence or presence of chloroquine (100 μM) were blotted with antibodies to ErbB2, p130Cas, LC3 and GAPDH as loading control. Right panel: Histograms show ErbB2 levels, normalized to GAPDH. Bars represent the means ± SEM of five independent experiments (ns: not significant; **p < 0.01; ***p < 0.001). (**B**) Left panel: BT474 cells as in (A) were treated with 100 μM chloroquine for 6 hours. Cell lysates were blotted with antibodies against ErbB2, p130Cas, LC3 and Actin as loading control. Right panel: Histograms show ErbB2 levels, normalized to Actin. Bars represent the means ± SEM of four independent experiments (ns: not significant; ***p < 0.001).

To demonstrate that the p130Cas expression can interfere with the autophagic degradation of ErbB2, Ctr, p130Cas silenced and overexpressing BT474 cells were starved and cultured for 6 hours in presence of HBSS (Hank's balanced salt solution) to induce autophagy alone or in combination with Chloroquine, a drug that arrests the latter step of autophagy, resulting in the failure of the autophagy process [24]. Cell lysates from control, p130Cas silenced and overexpressing cells were collected and western blot analysis performed. The results shown in Figure 3A (left and right panels) confirm that in untreated cells, silencing of p130Cas is sufficient to induce ErbB2 downregulation by autophagy as demonstrated by LC3-II upregulation. In addition, the induction of autophagy by HBSS treatment leads to approximately 50% reduction of ErbB2 in Ctr cells compared to untreated cells demonstrating that autophagy is implicated in the degradation of ErbB2. The effectiveness of HBSS treatment to trigger the autophagic flux is confirmed by the presence of the lipidated form of LC3. Most interestingly, in p130Cas overexpressing cells the HBSS treatment does not significantly affect ErbB2 expression levels indicating that the overexpression of p130Cas renders ErbB2 less sensitive to autophagy degradation. Moreover, the concomitant treatment of HBSS and Chloroquine results in the re-establishment of ErbB2 expression in p130Cas silenced cells upon autophagy inhibition (Figure 3A) and the concomitant accumulation of LC3-II expression, confirming that absence of p130Cas favors ErbB2 degradation through autophagy.

Accordingly, as shown in Figure 3B (left and right panels), the treatment with Chloroquine (100 μM) was sufficient to restore ErbB2 levels in both control and p130Cas silenced cells, while in p130Cas overexpressing cells did not result in a significant change in the amount of ErbB2 expression. Therefore, these results indicate that blocking the autophagic flux, as demonstrated by the increased expression of the lipidated form of LC3, rescues ErbB2 expression in both control and p130Cas silenced cells.

### p130Cas-dependent ErbB2 degradation by autophagy is regulated by mTOR activity

It is known that class I and class III of phosphatidylinositol 3-kinase (PI3K) can regulate autophagy in different ways. In cancer cells the growth factor receptor-induced activation of class I PI3K/AKT/mTOR axis, inhibits autophagy. Conversely, class III PI3K activity is required for the sequestration of cytoplasmic material that characterizes the autophagic process [25, 26].

In addition, we have previously demonstrated that p130Cas overexpression in ErbB2 transformed cells leads to the activation of mTOR [17]. Therefore, to identify which autophagy signaling pathways were specifically involved in p130Cas-mediated ErbB2 degradation, we treated control, p130Cas silenced and overexpressing BT474 cells for 16 hours with the mTOR activator MHY1485 (2 μM) to block autophagy [27] and with mTOR inhibitor rapamycin (100 nM) to induce autophagy.

**Figure 4:**
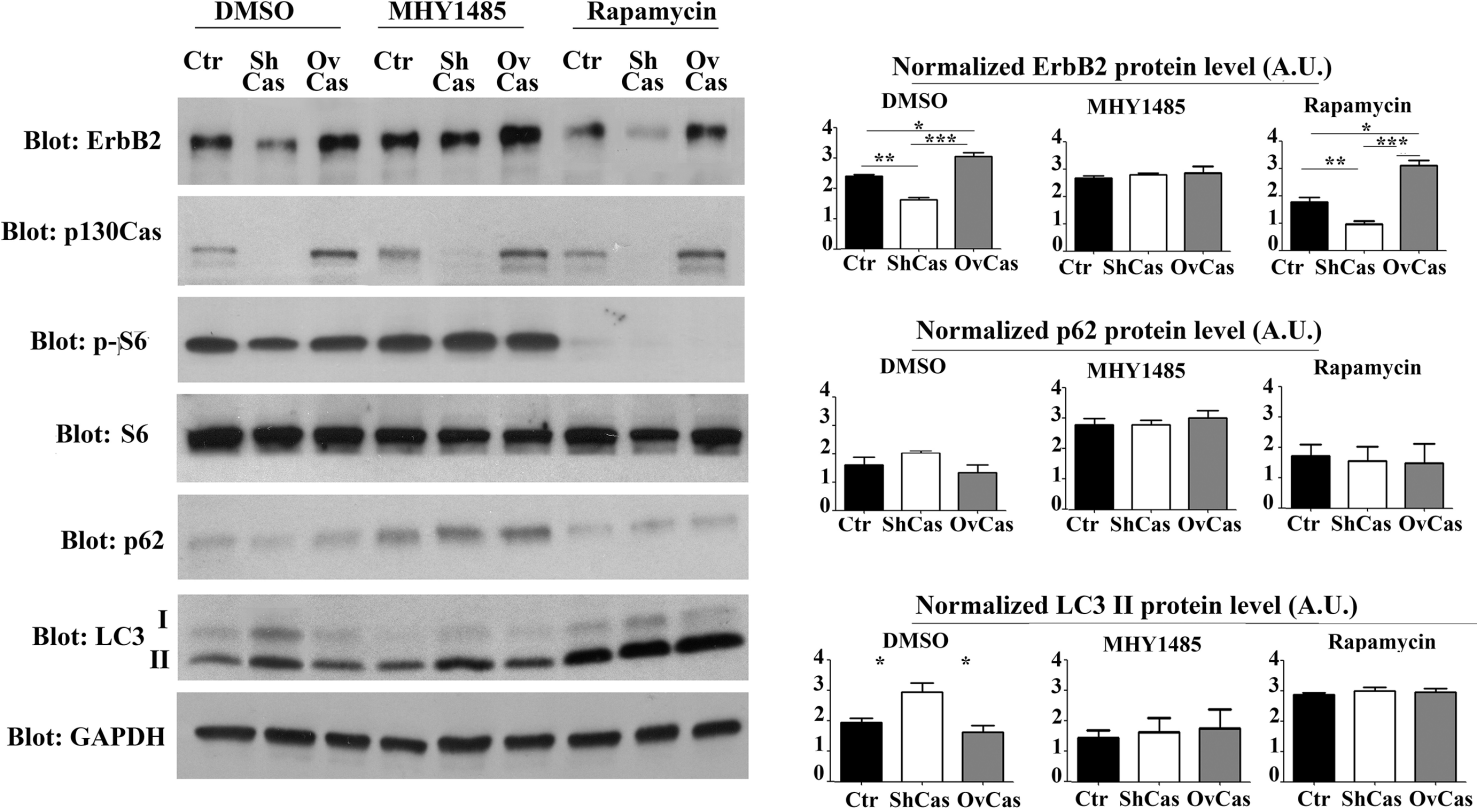
Inhibition of autophagy is sufficient to rescue p130Cas-dependent ErbB2 expression Left panel: BT474 silenced for p130Cas, overexpressing p130Cas and control cells were treated for 16 hours with mTOR activator MHY1485 (2 μM), with mTOR inhibitor Rapamycin (100 mM) and DMSO as control. Protein extracts were then blotted with antibodies against ErbB2, p130Cas, phospho-S6, S6, p62, LC3 and GAPDH as loading control. Right panel: Histograms show ErbB2, p62 and LC3 II levels, normalized to GAPDH. Bars represent the means ± SEM of five independent experiments (*p < 0.05; **p < 0.01; ***p < 0.001).

The results in Figure 4 indicate that treatment with Rapamycin in p130Cas silenced cells increased ErbB2 degradation. As expected, LC3-II expression was present following p130Cas silencing in control cells and was strongly upregulated in cells treated with Rapamycin. Conversely, experiments performed by treating cells with the mTOR activator, indicate that the resulting inhibition of autophagy is sufficient to reverse ErbB2 levels in p130Cas silenced BT474 cells, further confirming the key role of p130Cas in affecting signaling leading to autophagy that is instrumental for ErbB2 degradation. Importantly, p130Cas overexpressing cells were less prone to undergo autophagic degradation induced by Rapamycin. The inhibition of autophagy in cells treated with the mTOR activator is supported by low levels of LC3 expression and increased p62/sequestosome-1 protein expression. The same results were obtained by using the inhibitor of class III PI3K wortmannin, known to inhibit autophagy (data not shown) [25]. These data show that ErbB2 degradation by autophagy depends on p130Cas expression levels, with low levels of p130Cas sensitizing ErbB2 to authophagy degradation and high p130Cas levels protecting it from degradation.

### p130Cas protection of ErbB2 from ubiquitination

**Figure 5:**
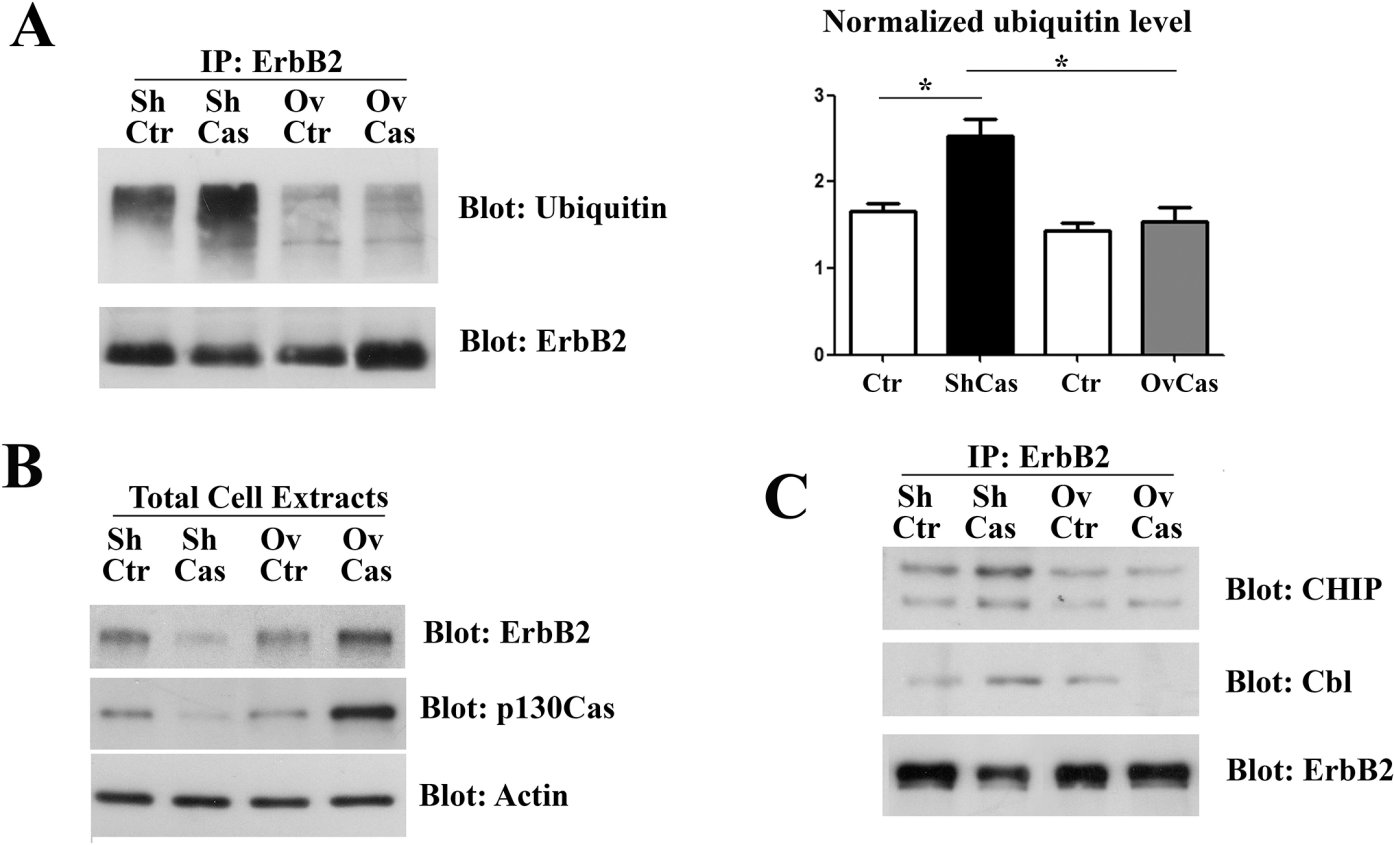
p130Cas expression influences ErbB2 ubiquitination (**A**) Left panel: Cell extracts from control (Ctr), p130Cas silenced and p130Cas overexpressing BT474 cells were immunoprecipitated with ErbB2 antibodies, followed by immunoblotting against Ubiquitin and ErbB2 antibodies. Right panel: Histograms show the levels of ubiquitin, normalized to ErbB2. Bars represent the means ± SEM of three independent experiments (*p < 0.05; **p < 0.01; ***p < 0.001). (**B**) Cell extracts as in A were probed with antibodies to p130Cas, ErbB2 and normalized with Vinculin. (**C**) Cell extracts from control (Ctr), p130Cas silenced and p130Cas overexpressing BT474 cells were immunoprecipitated with ErbB2 antibodies and blotted with Cbl, CHIP and ErbB2 antibodies.

It has been recently demonstrated that ubiquitin can be a crucial element to target protein aggregates to selective autophagy [28]. Therefore, we speculated that p130Cas can modify ErbB2 sensitivity to autophagy degradation by affecting its ubiquitination status. Therefore, ErbB2 was immunoprecipitated from p130Cas silenced or overexpressing BT474 cells and the immunoprecipitates were probed with anti-ubiquitin antibodies. As shown in Figure 5A, greater amounts of ubiquitinated ErbB2 were observed upon p130Cas silencing compared to control cells. In addition, higher levels of p130Cas were associated with lower ubiquitination of ErbB2. Western blotting analysis on the same cell extracts were performed to verify changes in ErbB2 level after modulation of p130Cas (Figure 5B).

These results indicate that the presence or absence of p130Cas can affect the ubiquitination of ErbB2. Since CHIP and Cbl E3 ligases have been reported to associate with ErbB2 and to mediate its ubiquitination [29–31] and we have previously demonstrated that p130Cas immunoprecipitates with ErbB2 ([15] and Supplementary Figure 5), we tested whether the observed differences of ErbB2 ubiquitination in presence or absence of p130Cas can be due to an impaired accessibility of ErbB2 by its E3 ligases. To this end, ErbB2 immunoprecipitates from BT474 cells in which p130Cas was silenced or overexpressed were probed with Cbl and CHIP antibodies. The results show that both CHIP and Cbl association to ErbB2 is highly increased in p130Cas silenced cells (Figure 5C), indicating that the binding of p130Cas to ErbB2 is sufficient to interfere with its polyubiquitination by reducing the binding of E3 enzymes, pointing out an uncovered chaperone function for p130Cas.

### p130Cas sustains ErbB2 stability and resistance to trastuzumab

**Figure 6:**
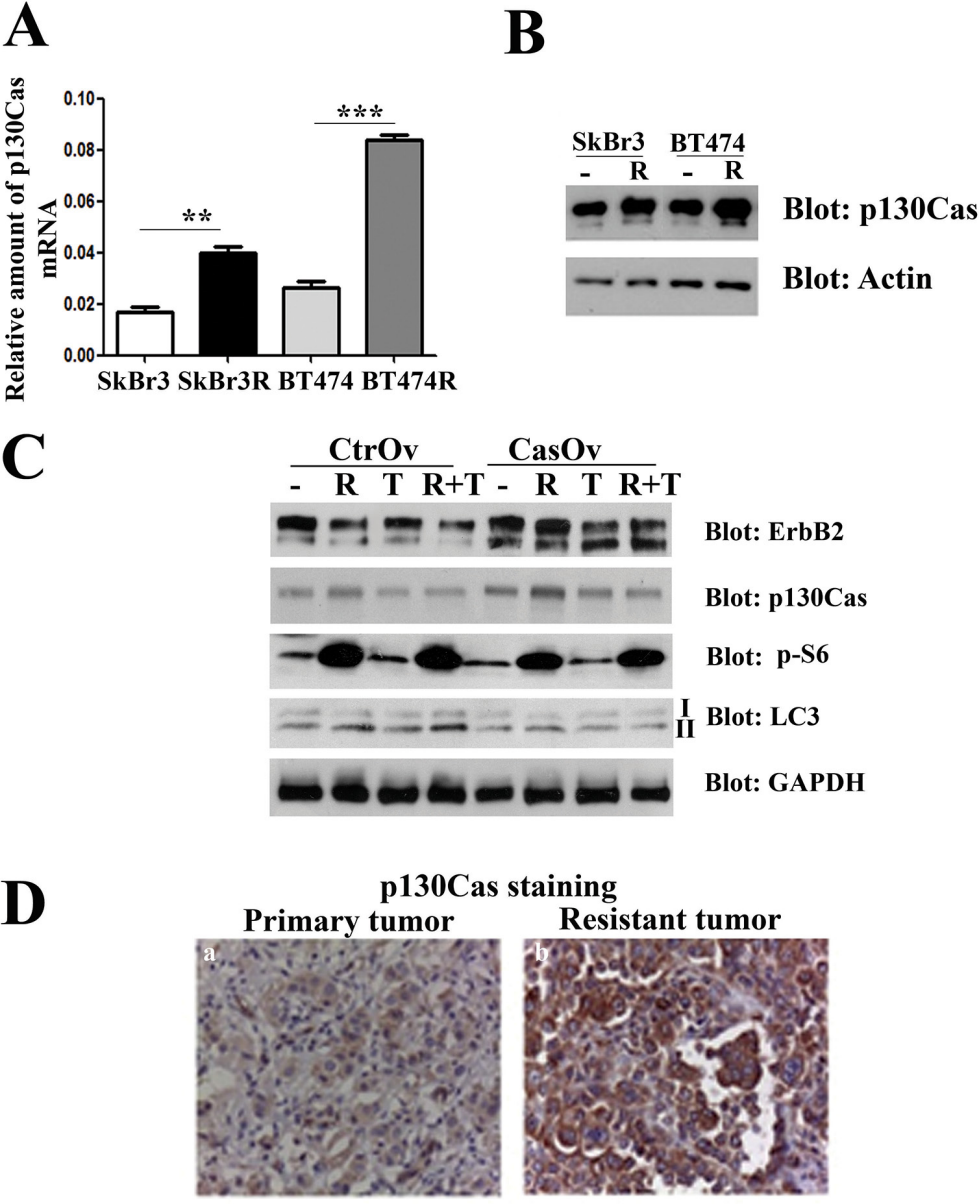
p130Cas overexpression promotes resistance to trastuzumab (**A**) Quantification of p130Cas mRNA by qRT PCR in wild type SKBR3 (SKBr3), in trastuzumab resistant SKBR3 (SKBr3R), in BT474 wild type (BT474) and in trastuzumab resistant BT474 cells (BT474R). The 18S housekeeping gene was used as an internal control for data normalization. (**B**) Cell extracts from trastuzumab sensitive and resistant SKBR3 and BT474 cells were probed with p130Cas antibodies and normalized with Actin. (**C**) Cell extracts from p130Cas overexpressing BT474 cells and relative control cells untreated or treated with 100 nM Rapamycin (R), 10 μM/ml trastuzumab (T) alone or in combination (R + T) were blotted with antibodies against ErbB2, p130Cas, phospho-S6, LC3 and GAPDH as loading control. (**D**) Representative images of p130Cas expression in primary tumor at the time of diagnosis (left panel) and in relapsing trastuzumab resistant tumor (right panel) of patient n°2 (see Table 1).

Since activation of ErbB2 downstream signaling and increased ErbB2 stability are hallmarks of resistance to trastuzumab treatment [32], we hypothesized that p130Cas can be a mediator of trastuzumab resistance. To examine whether p130Cas levels of expression is involved in trastuzumab resistance, experiments were performed in ErbB2 overexpressing BT474 and SKBR3 made resistant to trastuzumab [33]. Sensitive and resistant cells were first compared for levels of p130Cas mRNA and protein levels by performing quantitative real-time PCR and western blot analysis. The results indicate that transcription of p130Cas mRNA and its expression is upregulated in resistant SKBR3R and BT474R cells compared to wt cells suggesting a functional role of p130Cas in the acquired resistance to trastuzumab (Figure 6A and 6B). To assess whether the increased stability of ErbB2 following p130Cas-dependent inhibition of autophagy is a possible mechanism to induce resistance to trastuzumab, p130Cas overexpressing BT474 cells and relative controls were treated for 24 hours with rapamicyn (100 nM) and trastuzumab (10 μM) alone and/or in combination. As shown in Figure 6C, the treatment of cells with rapamycin and trastuzumab does not affect significantly the expression of ErbB2, whereas the combination of treatments results in downregulation of ErbB2, confirming what previously observed in preclinical studies using mTOR inhibitor and trastuzumab alone or in combination [34, 35]. Notably, the overexpression of p130Cas, as expected, is sufficient to induce the expression of ErbB2 and to prevent its downregulation in the combined treatment observed in control cells. These results indicate that the overexpression of p130Cas can be one important factor that contributes to trastuzumab resistance.

To further assess the involvement of p130Cas in clinical resistance to trastuzumab, we evaluated the correlation between p130Cas expression and failure to trastuzumab-based therapy in ErbB2 positive breast cancer patients. Therefore, we assessed the expression of p130Cas in ErbB2 positive primary tumors of 11 patients and in the relapsing trastuzumab resistant counterparts. As shown in Table 1, 8 out of 11 tumors showed an increase in p130Cas expression at the time of progression. Moreover, 3 out of 11 tumors were already 2+/3+ for p130Cas at first diagnosis of breast cancer and they retained this high p130Cas positivity after trastuzumab treatment (Figure 6D). These data suggest that high levels of p130Cas may promote acquired resistance to trastuzumab therapy in ErbB2 positive breast cancer.

**Table 1:** Grade of p130Cas expression in primary and relapsing breast cancer patients before and after trastuzumab treatment

Case N°	Stage	Histology	Mol. Subtype	Surgery at diagnosis	p130Cas (IHC before trastuzumab	p130Cas (IHC) after trastuzumab
1	III	CDI	HER2-Enriched	Yes	2+	3+
2	III	CDI	HER2-Enriched	Yes	1+	3+
3	III	CLI	HER2-Enriched	Yes	1+	3+
4	III	CDI	HER2-Enriched	Yes	3+	3+
5	III	CDI	HER2-Enriched	Yes	2+	3+
6	III	CDI	Luminal-HER2	Yes	1+	3+
7	III	CDI	Luminal-HER2	Yes	2+	3+
8	IV	CDI	Luminal-HER2	Not	3+	3+
9	I	CLI	HER2-Enriched	Yes	1+	2+
10	III	CDI	HER2-Enriched	Yes	2+	3+
11	III	CDI	HER2-Enriched	Yes	2+	2+

CDI: Ductal Infiltrating Carcinoma; CLI: Lobular Infiltrating Carcinoma; IHC: immunohistochemistry.

Overall our results indicate that the modulation of p130Cas expression correlates with alterations of ErbB2 expression, and in particular we demonstrated that p130Cas protects ErbB2 from autophagy degradation. This protection from degradation points out p130Cas as a crucial player in ErbB2 resistance to targeted therapy.

## DISCUSSION

Here we demonstrate for the first time that the adaptor protein p130Cas can act as a protecting agent against ErbB2 degradation. Consistently, we show that lowering p130Cas expression is sufficient to get ErbB2 degraded while p130Cas overexpression increased ErbB2 stability at the cell membrane. Our data also show that p130Cas-dependent ErbB2 degradation occurs preferentially by autophagy rather than by proteasome. Interestingly, we have demonstrated that the presence of p130Cas can impair the binding of the ErbB2 with its E3 ligases CHIP and Cbl, resulting in a defective ubiquitination.

We have previously extensively shown that p130Cas supports and amplifies ErbB2 downstream signaling pathways both *in vivo* and *in vitro* promoting tumorigenesis and progression [14–17]. Here we describe for the first time one possible mechanism through which p130Cas can affect ErbB2 tumorigenesis. Our present data indicate that p130Cas by binding to ErbB2, stabilizes the receptor preventing its ubiquitinylation and subsequent degradation by selective autophagy. Indeed p130Cas by binding to ErbB2 does not allow the association with CHIP and Cbl E3 ligases, possibly due to steric hindrance. Conversely, low levels of p130Cas allows the binding of CHIP and Cbl E3 ligases to ErbB2 promoting its degradation by autophagy.

It was previously inferred that ErbB2 receptor is resistant to down-regulation by endocytosis, probably because of its association to Hsp90 chaperone that might prevent due to steric hindrance the binding of E3 ligase to ErbB2 resulting in impaired ubiquitinylation and degradation [23]. Indeed Hsp90 inhibitors, such as geldamycin, induces a rapid ubiquitin-dependent degradation of the receptor [8, 9, 20, 36]. We can speculate that p130Cas mirrors in ErbB2 positive breast cancer the function of Hsp90, protecting ErbB2 from degradation but at the same time, assembling a signaling platform that sustains and reinforces breast cancer growth, migration and invasion.

Our data also points out that lowering p130Cas is sufficient to induce ErbB2 degradation that preferentially occurs by autophagy rather than by proteasome.

Interestingly, it has been speculated that the docking of proteins to be degraded to the ubiquitin-proteasome system (UPS) or to the autophagy—lysosomal pathway depends on the differential attachment of ubiquitin moieties. The association with K48-linked polyUb chains will represent the recognition signal for proteasome degradation, whereas the K63-linked chains may preferentially target substrates to degradation by autophagy [23, 28, 37, 38].

In parallel, it has been recently proposed in SKBR3 breast cancer cells that ErbB2 degradation is proteasome independent and that the conjugation of K63-linked polyubquitin chains to ErbB2 might be relevant to target its degradation by the autophagy-lysosomal system [21, 23]. On the basis of these data we can speculate that in the absence of p130Cas the observed ErbB2 degradation by autophagy might be dependent by an increased amount of K63 linkages that in turn are recognized by the autophagy receptors and finally degraded. Further investigations are needed to verify this hypothesis and to identify the molecular players involved in p130Cas-dependent ErbB2 degradation and/or stability.

Nevertheless, the translational importance of the levels of p130Cas expression in the regulation of ErbB2 stability is evident. Indeed, the p130Cas silencing in a therapeutic setting will contribute to ErbB2 degradation and thereby to limitations of its oncogenic properties, whereas the overexpression of p130Cas by protecting ErbB2 from autophagy can be implicated in resistance to trastuzumab treatment. Our data indeed indicate that high levels of p130Cas expression inversely correlate with ErbB2 sensitivity to trastuzumab and the induction of autophagy is not sufficient to promote its degradation. The mechanism through which p130Cas mediates resistance to trastuzumab might rely on the increased ErbB2 stability to the cell membrane. However, it has been reported that increased autophagosome formation in trastuzumab resistant cells preserves breast cancer cell survival [39]. These opposite data can be reconciled by the fact that overexpression of p130Cas drives resistance to trastuzumab by increasing ErbB2 stability and by blocking the receptor ubiquitination and thus its degradation, regardless of activation of downstream autophagy flux.

Our and others previous and current results indicate that high levels of p130Cas are hallmarks of breast cancer progression, invasion, metastatization and resistance [11, 13, 40]. However, the mechanisms through which p130Cas expression is upregulated in breast cancer still remain an open question.

In conclusion, we provided evidence that p130Cas overexpression prevents ErbB2 degradation by autophagy. The resulting increased stabilization of ErbB2 by p130Cas might be the crucial event driving breast cancer progression and resistance, strengthening the relevance of p130Cas as an unfavorable prognostic marker and a putative therapeutic target to overcome resistance to trastuzumab based treatment in ErbB2 positive breast cancers.

## MATERIALS AND METHODS

### Antibodies

p130Cas mAbs have been previously described [41]. ErbB2 NCL-L-Cb11 mAbs were purchased from Novocastra (Leica Microsystems Srl, Germany). An additional mAb directed to the cytoplasmic domain of ErbB2 were prepared in our laboratory, by immunizing mice with a recombinant protein encompassing amino acids 1031–1160 of rat ErbB2 cDNA sequence. mAbs to Vinculin were from Millipore (Billerica, MA, USA). Antibodies to c-Src, p-Tyr PY99, Actin, Cbl and CHIP were from Santa Cruz Biotechnologies (Palo Alto, CA, USA). pTyr416 c-Src and Beclin-1 antibodies were from Cell Signaling (Beverly, MA, USA). Mono- and polyubiquitinylated conjugates antibody was from Enzo Life Sciences (Farmingdale, NY). LC3 polyclonal antibody was from Thermo Scientific Pierce Antibodies (Rockford, Illinois). Secondary antibodies conjugated with peroxidase were from Sigma-Aldrich (St. Louis, MO, USA).

### Cell lines

SKBR3 cells were cultured in McCoy's 5A–15% FBS and BT474 cell lines were cultured in DMEM-F12 with 10% FBS. SKBR3 and BT474 resistant cells were respectively cultured in RPMI-1640 10% FBS and DMEM 10% FBS. SKBR3 and BT474 made resistant to trastuzumab were generated by Dr. Valabrega as described in [33].

### Proteasome and autophagy experiments

For proteasome inhibition experiments, MG132 (CAS-133407-82-6, Calbiochem, Darmstadt, Germany) was directly added into medium at a concentration of 0.5, 2 and 6 μM for 16 hours. Cycloheximide was used at 100 mg/ml and purchased from Sigma (Sigma-Aldrich, St. Louis, MO, USA). For autophagy induction cells were cultured in Hank's balanced salt solution (HBSS, Invitrogen Life Technologies, Carlsbad, CA, USA) for 2, 4 and 6 hours. For autophagy pharmacological inhibition, cells were treated with the PI3-kinase inhibitor, wortmannin (Enzo Life Sciences, Farmingdale, NY) at 100 nM and with chloroquine (Sigma-Aldrich, St. Louis, MO, USA), an inhibitor of autophagosome formation at 100 μM for 16 hours. For mTOR inhibition cells were treated with rapamycin (Cell Signaling Technology, Beverly, MA) at 100 nM for 16 hours. For resistance experiments, cells were treated for 20 hours with trastuzumab (10 microg/ml) or Rapamycin (100 nM) or with combination of both.

### Lentivirus generation

A pLKO.1 lentiviral vector carrying a shRNA directed to human p130Cas (p130Cas shRNA) was selected in the pLKO.1 target gene shRNA set (clone ID TRCN0000115985), purchased from Open Biosystem (Huntsville, AL, USA, http://www.thermoscientificbio.com). pLKO.1 scramble shRNA vector (Addgene, Cambridge, MA, USA, http://www.addgene.com) was used as negative control. Lentiviral particles were generated and concentrated by ultracentrifugation (50,000 g, 2 hours). BT474 and SKBR3 cells were infected with the lentiviral p130Cas-shRNA (sh-p130Cas) and scramble-shRNA (Ctr). Puromycin (Sigma) (0.5 mg/ml) was added 24 hours after infection with PLKO.1 vectors as described in [42]. For p130Cas expression, human p130Cas cDNA, mouse p130Cas cDNA were cloned into pCCL lentiviral vector, and viral particles production was performed as described in [42].

### Immunobloting and immunoprecipitation analysis

Cells were extracted with RIPA buffer (1% Triton X-100, 0,1% SDS, 1% sodium deoxycholate, 150 mM NaCl, 50 mM Tris-HCl ph 7, 0.4 mM Na3VO4, inhibitor mix). Cell lysates were centrifuged at 13,000 g for 10 minutes and the supernatants were collected and assayed for protein concentration with the Bio-Rad protein assay method (Biorad, Hercules, CA, USA). Proteins were run on SDS-PAGE under reducing conditions. Following SDS-PAGE, proteins were transferred to nitrocellulose, incubated with specific antibodies and then detected with peroxidase conjugated secondary antibodies and chemoluminescent ECL reagent. When appropriate, the nitrocellulose membranes were stripped according to manufacturers' recommendations and re-probed. Densitometric analysis was performed using the GS 250 Molecular Imager (Biorad).

For immunoprecipitation assay, 1 mg of cell extracts were immunoprecipitated with ErbB2 antibody and then probed for specific antibodies.

For ubiquitination experiments, 10 mg of cell extracts were immunoprecipitated with ErbB2 antibody. After blotting, the membrane were pre-incubated with denaturing buffer (6 M guanidine-HCl, 20 mM Tris-HCl ph 7.5, 5 mM b-mercapto-ethanol, 1 mM PMSF) for 30′ at 4°C and washed in PBS buffer. Then the membrane was blocked with 5% BSA in TBS buffer for 6 hours at room temperature and incubated overnight at 4°C with the anti-ubiquitin antibody.

### RNA isolation and qRT-PCR for mRNA detection

Total RNA was isolated from cells using TRIzol^™^ Reagent (Invitrogen Life Technologies, Carlsbad, CA, USA). 1 μg of DNAse-treated RNA (RQ1 RNase-Free DNase kit, Promega, Madison, WI, USA) was retrotranscribed with High Capacity cDNA Reverse Transcription Kit (Invitrogen Life Technologies, Carlsbad, CA, USA). Quantitative PCR was performed on an Applied Biosystems, 7900HT Fast Real-Time PCR System (standard settings) using the Universal Probe Library system (Roche Italia, Monza, Italy) and Platinum^™^ Quantitative PCR SuperMix-UDG (Invitrogen Life Technologies, Carlsbad, CA, USA). Results were analyzed with the 2^−ΔΔCt^ method using the 18 S rRNA predeveloped TaqMan assay (Invitrogen Life Technologies, Carlsbad, CA, USA) as an internal control. The median expression across samples was used as calibrator.

### *In vitro* cell assays

For proliferation assay, MTT (4,5 dimethyl-2-yl 2,5-diphenyl tetrazolium bromide) from Sigma Chemical Co. (St. Louis, MO, USA) was performed on SKBR3 and BT474 upon treatment with freshly added trastuzumab (Herceptin) (2 μg/ml) for 4 days. For FACS analysis cells were stained with ErbB2 mAb and with Alexa 488 secondary antibody (Invitrogen Life Technologies, Carlsbad, CA, USA). Alexa 488 emission was detected in the green channel (525 nm) following excitation by a 488 nm laser on a FACS Calibur cytometer (Becton, Dickinson and Company, Franklin Lakes, NJ, USA).

### Immunohistochemistry procedures

Human investigations were performed with informed consent and were preceded by local institutional review board approval.

Samples were routinely fixed in 10% formaldehyde buffer (pH 7.4) for 24 hrs, paraffin-embedded, and processed for immunohistochemical analysis. Slides were incubated with anti-p130Cas (Labvision Thermo Scientific) (1 microg/mL) for 1 hr at room temperature, after antigen retrieval (citrate buffer, at 98°C for 40 min). Staining was detected with EnVision System-HRP Labelled Polymer anti-mouse (DakoCytomation) and developed with the LiquidDAB Substrate Pack (BioGenex, San Ramon, CA, USA). Nuclei were counterstained with Mayer hemallum. Images were taken using a Leica DM 2000 microscope.

### Statistical analysis

The results are representative of at least three independent experiments performed in triplicate and are expressed as the means ± s.e.m. Statistical analysis of the data was performed using a Student's *t*-test.

## SUPPLEMENTARY MATERIALS FIGURES



## References

[1] Baselga J (2010). Treatment of HER2-overexpressing breast cancer. Ann Oncol.

[2] Emde A, Kostler WJ, Yarden Y (2012). Therapeutic strategies and mechanisms of tumorigenesis of HER2-overexpressing breast cancer. Crit Rev Oncol Hematol.

[3] Valabrega G, Montemurro F, Aglietta M (2007). Trastuzumab: mechanism of action, resistance and future perspectives in HER2-overexpressing breast cancer. Ann Oncol.

[4] Mohd Sharial MS, Crown J, Hennessy BT (2012). Overcoming resistance and restoring sensitivity to HER2-targeted therapies in breast cancer. Ann Oncol.

[5] Muthuswamy SK (2011). Trastuzumab resistance: all roads lead to SRC. Nat Med.

[6] Pohlmann PR, Mayer IA, Mernaugh R (2009). Resistance to Trastuzumab in Breast Cancer. Clin Cancer Res.

[7] Valabrega G, Montemurro F, Sarotto I, Petrelli A, Rubini P, Tacchetti C, Aglietta M, Comoglio PM, Giordano S (2005). TGFalpha expression impairs Trastuzumab-induced HER2 downregulation. Oncogene.

[8] Lerdrup M, Hommelgaard AM, Grandal M, van Deurs B (2006). Geldanamycin stimulates internalization of ErbB2 in a proteasome-dependent way. J Cell Sci.

[9] Raja SM, Clubb RJ, Bhattacharyya M, Dimri M, Cheng H, Pan W, Ortega-Cava C, Lakku-Reddi A, Naramura M, Band V, Band H (2008). A combination of Trastuzumab and 17-AAG induces enhanced ubiquitinylation and lysosomal pathway-dependent ErbB2 degradation and cytotoxicity in ErbB2-overexpressing breast cancer cells. Cancer Biol Ther.

[10] Defilippi P, Di Stefano P, Cabodi S (2006). p130Cas: a versatile scaffold in signaling networks. Trends Cell Biol.

[11] Cabodi S, del Pilar Camacho-Leal M, Di Stefano P, Defilippi P (2010). Integrin signalling adaptors: not only figurants in the cancer story. Nat Rev Cancer.

[12] Nikonova AS, Gaponova AV, Kudinov AE, Golemis EA (2014). CAS proteins in health and disease: an update. IUBMB Life.

[13] Tornillo G, Defilippi P, Cabodi S (2014). Cas proteins: dodgy scaffolding in breast cancer. Breast Cancer Res.

[14] Cabodi S, Tinnirello A, Di Stefano P, Bisaro B, Ambrosino E, Castellano I, Sapino A, Arisio R, Cavallo F, Forni G, Glukhova M, Silengo L, Altruda F (2006). p130Cas as a new regulator of mammary epithelial cell proliferation, survival, and HER2-neu oncogene-dependent breast tumorigenesis. Cancer Res.

[15] Cabodi S, Tinnirello A, Bisaro B, Tornillo G, del Pilar Camacho-Leal M, Forni G, Cojoca R, Iezzi M, Amici A, Montani M, Eva A, Di Stefano P, Muthuswamy SK (2010). p130Cas is an essential transducer element in ErbB2 transformation. FASEB J.

[16] Pincini A, Tornillo G, Orso F, Sciortino M, Bisaro B, Leal Mdel P, Lembo A, Brizzi MF, Turco E, De Pitta C, Provero P, Medico E, Defilippi P (2013). Identification of p130Cas/ErbB2-dependent invasive signatures in transformed mammary epithelial cells. Cell Cycle.

[17] Tornillo G, Bisaro B, Camacho-Leal Mdel P, Galie M, Provero P, Di Stefano P, Turco E, Defilippi P, Cabodi S (2011). p130Cas promotes invasiveness of three-dimensional ErbB2-transformed mammary acinar structures by enhanced activation of mTOR/p70S6K and Rac1. Eur J Cell Biol.

[18] Brinkman A, van der Flier S, Kok EM, Dorssers LC (2000). BCAR1, a human homologue of the adapter protein p130Cas, and antiestrogen resistance in breast cancer cells. J Natl Cancer Inst.

[19] Ta HQ, Thomas KS, Schrecengost RS, Bouton AH (2008). A novel association between p130Cas and resistance to the chemotherapeutic drug adriamycin in human breast cancer cells. Cancer Res.

[20] Bertelsen V, Stang E (2014). The Mysterious Ways of ErbB2/HER2 Trafficking. Membranes (Basel).

[21] Marx C, Yau C, Banwait S, Zhou Y, Scott GK, Hann B, Park JW, Benz CC (2007). Proteasome-regulated ERBB2 and estrogen receptor pathways in breast cancer. Mol Pharmacol.

[22] Zhao M, Vuori K (2011). The docking protein p130Cas regulates cell sensitivity to proteasome inhibition. BMC Biol.

[23] Marx C, Held JM, Gibson BW, Benz CC (2010). ErbB2 trafficking and degradation associated with K48 and K63 polyubiquitination. Cancer Res.

[24] Kimura T, Takabatake Y, Takahashi A, Isaka Y (2013). Chloroquine in cancer therapy: a double-edged sword of autophagy. Cancer Res.

[25] Kondo Y, Kanzawa T, Sawaya R, Kondo S (2005). The role of autophagy in cancer development and response to therapy. Nat Rev Cancer.

[26] Mizushima N, Yoshimori T, Levine B (2010). Methods in mammalian autophagy research. Cell.

[27] Choi YJ, Park YJ, Park JY, Jeong HO, Kim DH, Ha YM, Kim JM, Song YM, Heo HS, Yu BP, Chun P, Moon HR, Chung HY (2012). Inhibitory effect of mTOR activator MHY1485 on autophagy: suppression of lysosomal fusion. PLoS One.

[28] Kirkin V, McEwan DG, Novak I, Dikic I (2009). A role for ubiquitin in selective autophagy. Mol Cell.

[29] Klapper LN, Waterman H, Sela M, Yarden Y (2000). Tumor-inhibitory antibodies to HER-2/ErbB-2 may act by recruiting c-Cbl and enhancing ubiquitination of HER-2. Cancer Res.

[30] Xu W, Marcu M, Yuan X, Mimnaugh E, Patterson C, Neckers L (2002). Chaperone-dependent E3 ubiquitin ligase CHIP mediates a degradative pathway for c-ErbB2/Neu. Proc Natl Acad Sci U S A.

[31] Zhou P, Fernandes N, Dodge IL, Reddi AL, Rao N, Safran H, DiPetrillo TA, Wazer DE, Band V, Band H (2003). ErbB2 degradation mediated by the co-chaperone protein CHIP. J Biol Chem.

[32] Bailey TA, Luan H, Clubb RJ, Naramura M, Band V, Raja SM, Band H (2011). Mechanisms of Trastuzumab resistance in ErbB2-driven breast cancer and newer opportunities to overcome therapy resistance. J Carcinog.

[33] Valabrega G, Capellero S, Cavalloni G, Zaccarello G, Petrelli A, Migliardi G, Milani A, Peraldo-Neia C, Gammaitoni L, Sapino A, Pecchioni C, Moggio A, Giordano S (2011). HER2-positive breast cancer cells resistant to trastuzumab and lapatinib lose reliance upon HER2 and are sensitive to the multitargeted kinase inhibitor sorafenib. Breast Cancer Res Treat.

[34] Miller TW, Forbes JT, Shah C, Wyatt SK, Manning HC, Olivares MG, Sanchez V, Dugger TC, de Matos Granja N, Narasanna A, Cook RS, Kennedy JP, Lindsley CW (2009). Inhibition of mammalian target of rapamycin is required for optimal antitumor effect of HER2 inhibitors against HER2-overexpressing cancer cells. Clin Cancer Res.

[35] Zhu Y, Zhang X, Liu Y, Zhang S, Liu J, Ma Y, Zhang J (2012). Antitumor effect of the mTOR inhibitor everolimus in combination with trastuzumab on human breast cancer stem cells *in vitro* and *in vivo*. Tumour Biol.

[36] Pedersen NM, Madshus IH, Haslekas C, Stang E (2008). Geldanamycin-induced down-regulation of ErbB2 from the plasma membrane is clathrin dependent but proteasomal activity independent. Mol Cancer Res.

[37] Olzmann JA, Chin LS (2008). Parkin-mediated K63-linked polyubiquitination: a signal for targeting misfolded proteins to the aggresome-autophagy pathway. Autophagy.

[38] Shaid S, Brandts CH, Serve H, Dikic I (2013). Ubiquitination and selective autophagy. Cell Death Differ.

[39] Vazquez-Martin A, Oliveras-Ferraros C, Menendez JA (2009). Autophagy facilitates the development of breast cancer resistance to the anti-HER2 monoclonal antibody trastuzumab. PLoS One.

[40] Tikhmyanova N, Golemis EA (2011). NEDD9 and BCAR1 negatively regulate E-cadherin membrane localization, and promote E-cadherin degradation. PLoS One.

[41] Cabodi S, Moro L, Baj G, Smeriglio M, Di Stefano P, Gippone S, Surico N, Silengo L, Turco E, Tarone G, Defilippi P (2004). p130Cas interacts with estrogen receptor alpha and modulates non-genomic estrogen signaling in breast cancer cells. J Cell Sci.

[42] Bisaro B, Montani M, Konstantinidou G, Marchini C, Pietrella L, Iezzi M, Galie M, Orso F, Camporeale A, Colombo SM, Di Stefano P, Tornillo G, Camacho-Leal MP (2012). p130Cas/Cyclooxygenase-2 axis in the control of mesenchymal plasticity of breast cancer cells. Breast Cancer Res.

