# Supplementary material for: p130Cas scaffold protein regulates ErbB2 stability by altering breast cancer cell sensitivity to autophagy
Source: Oncotarget. 2015 Dec 21;7(4):4442–53. doi: 10.18632/oncotarget.6710 (PMC4826217; doi:10.18632/oncotarget.6710)
Supplement: Supplementary file 1 [file oncotarget-07-4442-s001.pdf]

p130Cas scaffold protein regulates ErbB2 stability by altering breast cancer cell sensitivity to autophagy

Supplementary Materials

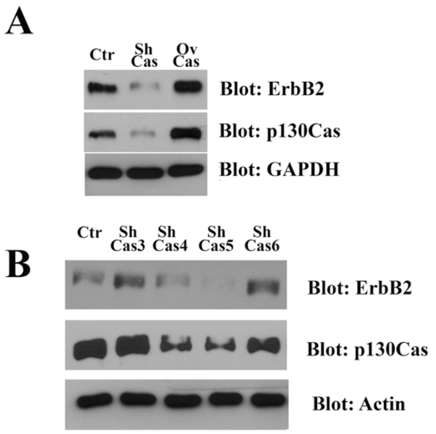

Supplementary Figure S1: Modulation of p130Cas expression specifically affects ErbB2 expression. (A) Total cell lysates of SKBR3 cells infected with lentiviral vectors to silence (Cas sh) or overexpress p130Cas (Cas over) were blotted with p130Cas and ErbB2 antibodies. GAPDH was used as loading control. **(B)** Total cell lysates of BT474 cells infected with lentiviral vectors carrying four putative silencing sequences of p130Cas were blotted with p130Cas and ErbB2 antibodies. GAPDH was used as loading control.

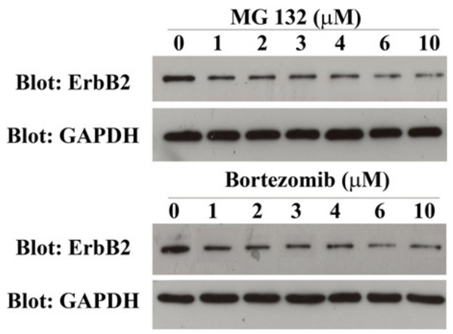

Supplementary Figure S2: Treatment with proteasome inhibitors promotes ErbB2 degradation. WT BT474 breast cancer cells were treated with different doses of the proteasome inhibitors MG132 (upper panel) or Bortezomib (lower panel) and total cell lysates were blotted with ErbB2 and GAPDH as loading control.

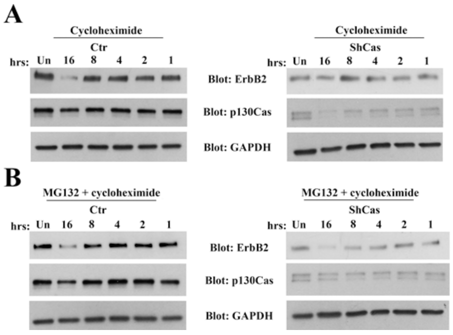

Supplementary Figure S3: p130Cas downregulation prompts ErbB2 degradation. (**A**) Extracts from control (Ctr) and p130Cas silenced BT474 cells (left and right panel, respectively) were treated for the indicated hours with Cycloheximide (100 µg/ml) and blotted with antibodies to ErbB2, p130Cas and GAPDH as loading control. (**B**) Cells as in (A) were treated for the indicated hours with Cycloeximide (100 µg/ml) and MG132 (2 µM) and total cell extracts were blotted with antibodies to ErbB2, p130Cas and GAPDH as loading control.

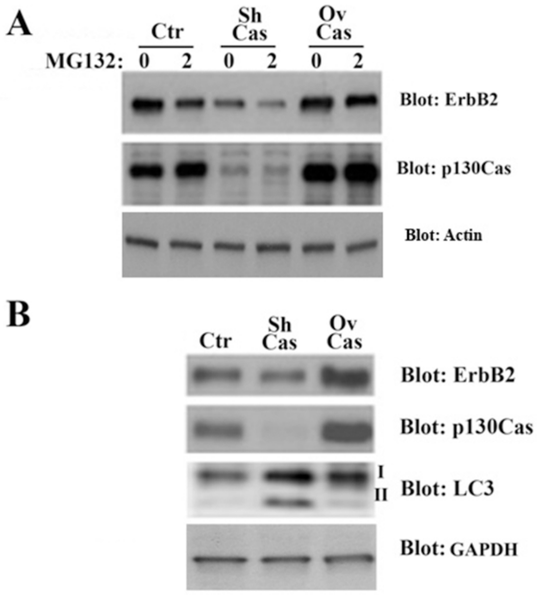

Supplementary Figure S4: p130Cas protects ErbB2 from autophagy-dependent degradation. (A) SKBR3 silenced for or overexpressing p130Cas and relative controls were treated for 16 hours with MG132 (2 µM). Protein extracts were then blotted with antibodies against ErbB2, p130Cas and actin for loading control. (**B**) Total cell lysates of SKBR3 cells infected with lentiviral vectors to silence (Cas sh) or overexpress p130Cas (Cas over) were blotted with ErbB2, p130Cas and LC3 antibodies. GAPDH was used as loading control.

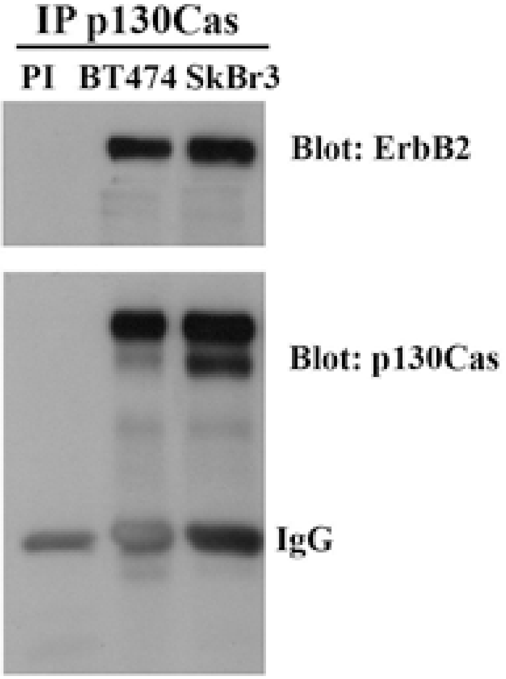

Supplementary Figure S5: p130Cas and ErbB2 co-immunoprecipitate. Cell extracts from control BT474 and SKBR3 cells were immunoprecipitated with isotype-specific antibodies (PI) or mAbs for p130Cas and blotted with ErbB2 and p130Cas antibodies.
